# Understanding the contribution of metabolism to *Mycobacterium tuberculosis* drug tolerance

**DOI:** 10.3389/fcimb.2022.958555

**Published:** 2022-08-22

**Authors:** Amanda N. Samuels, Erin R. Wang, Gregory A. Harrison, Joy C. Valenta, Christina L. Stallings

**Affiliations:** Department of Molecular Microbiology, Washington University School of Medicine, Saint Louis, MO, United States

**Keywords:** tuberculosis, antibiotics, tolerance, metabolism, granuloma, cholesterol, hypoxia

## Abstract

Treatment of *Mycobacterium tuberculosis* (*Mtb)* infections is particularly arduous. One challenge to effectively treating tuberculosis is that drug efficacy *in vivo* often fails to match drug efficacy *in vitro.* This is due to multiple reasons, including inadequate drug concentrations reaching *Mtb* at the site of infection and physiological changes of *Mtb* in response to host derived stresses that render the bacteria more tolerant to antibiotics. To more effectively and efficiently treat tuberculosis, it is necessary to better understand the physiologic state of *Mtb* that promotes drug tolerance in the host. Towards this end, multiple studies have converged on bacterial central carbon metabolism as a critical contributor to *Mtb* drug tolerance. In this review, we present the evidence that changes in central carbon metabolism can promote drug tolerance, depending on the environment surrounding *Mtb*. We posit that these metabolic pathways could be potential drug targets to stymie the development of drug tolerance and enhance the efficacy of current antimicrobial therapy.

## *Mtb* infections are recalcitrant to antibiotic therapy

Tuberculosis, an infection caused by the pathogen *Mycobacterium tuberculosis (Mtb)* is one of the leading causes of death world-wide by an infectious agent (WHO, 2021). Standard of care treatment for drug sensitive *Mtb* infections requires at least 6 months of antibiotic therapy with 4 or more antibiotics (WHO, 2017). Infection with *Mtb* mutants that are resistant to the frontline antibiotics isoniazid and rifampicin constituted approximately half a million tuberculosis cases in 2019 (WHO, 2021) and contributes to treatment failure (Chen et al., 2020). The treatment regimen for patients harboring drug resistant *Mtb* is even longer and more expensive than drug sensitive cases and has an increased risk of adverse side effects (Nahid et al., 2019; WHO, 2021; Ghazy et al., 2022). Overall, the emergence and prevalence of *Mtb* drug resistance threatens treatment efficacy globally.

In addition, treatment failure and relapse can occur even in the absence of drug resistance. Dating as far back as the 1950’s, it is documented that *Mtb* can be recovered from some patients after antibiotic treatment, with a fraction of these isolates remaining drug sensitive *in vitro* (Hobby, 1955; Wallace and Sutherland, 1955). In a 2014 study, 8% of patients that were treated with the standard of care isoniazid, rifampin, pyrazinamide, and ethambutol for 8 weeks followed by 18 weeks of isoniazid and rifampicin had an unfavorable outcome (Gillespie et al., 2014). The most common unfavorable outcome was relapse, which was differentiated from patients re-infected with another strain by using 24-locus mycobacterial-interspersed-repetitive-unit analysis to confirm that the strains isolated during relapse were the same as the primary infection (Gillespie et al., 2014). In this study, only 25% of the patients receiving the standard of care that relapsed after conversion to culture-negative status were suspected to have acquired drug resistance (Gillespie et al., 2014). Shortening the antibiotic regimen results in even further increased rates of treatment failure and relapse (Gillespie et al., 2014; Jindani et al., 2014). Another study collected serial *Mtb* isolates from tuberculosis patients that had relapsed infection after antibiotic treatment, where relapse was defined by paired isolates exhibiting 0-6 single nucleotide polymorphisms by whole genome sequencing (Bryant et al., 2013). In this study, all the relapsed *Mtb* isolates were drug sensitive *in vitro* (Bryant et al., 2013). Collectively, this data support that a reservoir of drug sensitive *Mtb* can persist in the host despite antibiotic therapy, contributing to treatment failure in some patients.

## Factors that contribute to *Mtb* surviving antibiotic treatment *in vivo*

Multiple factors have been identified that enable *Mtb* to persist in the host during antibiotic treatment without acquiring a drug resistance mutation. One factor is the pathology that develops within the lung during *Mtb* infection. During infection, the interaction between *Mtb* and the host immune response can result in the development of a granuloma, which is made up of host immune cells, *Mtb*, and tissue debris (Ehlers and Schaible, 2013). Antibiotic penetration into the granuloma can be limited based on the chemical properties of the antibiotic, which creates a challenge for efficient delivery of the antibiotic to the various sites where *Mtb* resides (Kjellsson et al., 2012; Prideaux et al., 2015; Sarathy et al., 2018). In addition, *Mtb* can reside within various compartments inside innate immune cells, which can impact antibiotic efficacy. For example, pyrazinamide preferentially accumulates and is maximally active against *Mtb* in acidified compartments within the macrophage (Santucci et al., 2021; Santucci et al., 2022).

In addition to the host response impacting antibiotic accessibility to *Mtb*, the pathogen itself changes its physiology in response to the host environment, resulting in phenotypic drug tolerance. Importantly, drug tolerance is different from drug resistance in that a drug tolerant population can survive in the presence of an antibiotic but cannot grow until the antibiotic pressure is removed, whereas a drug resistant population can both survive and replicate in the presence of an antibiotic. In unstressed axenic culture conditions, *Mtb* populations display a basal level of heterogeneity such that a subpopulation of bacteria is transiently tolerant to antibiotics (Aldridge et al., 2012; Manina et al., 2015; Rego et al., 2017). Because of this drug tolerant subpopulation, treatment with a bactericidal antibiotic, such as isoniazid or rifampicin, leads to a significant decrease in viable bacteria, but fails to sterilize the culture (Jain et al., 2016; Sukheja et al., 2017; Vilcheze et al., 2017). Some of the drug susceptibility heterogeneity results from *Mtb*’s asymmetric cell division (Aldridge et al., 2012; Rego et al., 2017). Deletion of the gene *lamA/mmpS3* leads to a loss of asymmetric cell elongation and cell size heterogeneity in *Mycobacterium smegmatis*, and an *Mtb lamA/mmpS3* mutant is more susceptible to killing by rifampicin and vancomycin, suggesting that asymmetric cell elongation and cell size heterogeneity contributes to the emergence of drug tolerant subpopulations (Rego et al., 2017). In addition, there are stochastic differences in gene expression within mycobacterial cultures that can affect antibiotic susceptibility. For example, mycobacteria exhibit stochastic variation in the expression of *katG*, which is required to activate the pro-drug isoniazid, leading to a small population of bacteria with transiently low *katG* expression that can survive exposure to isoniazid (Wakamoto et al., 2013).

The proportion of drug tolerant *Mtb* is higher *in vivo* when compared to the small population that exists at basal levels in unstressed axenic cultures. *Mtb* directly isolated from patient sputum samples exhibited a nearly 10-fold reduction in killing by streptomycin, isoniazid, ethambutol, or rifampicin in comparison to when those same isolates were passaged through normal culture conditions (Turapov et al., 2016). *Mtb* in caseum isolated from infected rabbit granulomas also exhibited a >100-fold increase in the minimum bactericidal concentration for rifampicin and isoniazid compared to *Mtb* growing *in vitro* (Sarathy et al., 2018). Therefore, the *Mtb* population at the site of infection is enriched for drug tolerant cells, indicating that the host environment causes the *Mtb* population to shift towards a more drug tolerant state. Understanding the mechanistic basis for this enhanced drug tolerance is essential for developing therapies that target the *Mtb* population that is recalcitrant to treatment.

## Stresses encountered in the host promote drug tolerance

During infection of macrophages, *Mtb* may be exposed to low pH, nitrosative stress, oxidative stress, osmotic changes, carbohydrate limitation, and cell envelope damage (Schnappinger et al., 2003; Tan et al., 2013; Larrouy-Maumus et al., 2016; Pisu et al., 2020). The environment within granulomas also poses additional stresses on *Mtb*, where granulomas can be hypoxic (Via et al., 2008), contain host factors that sequester iron (Kurthkoti et al., 2017), and harbor host enzymes that produce reactive oxygen species (Marakalala et al., 2016). Despite this harsh host environment, *Mtb* can survive due to its robust stress response capabilities. *Mtb* responds transcriptionally and metabolically to survive exposure to hypoxia (Wayne and Hayes, 1996), nitric oxide (Voskuil et al., 2003), reactive oxygen species (Voskuil et al., 2011), carbon limitation (Loebel et al., 1933; Betts et al., 2002; Gengenbacher et al., 2010), iron limitation (Kurthkoti et al., 2017), and low pH (Baker et al., 2014). Importantly, when exposed to stress *in vitro*, such as hypoxia, low pH, changes in osmolarity, or nutrient limitation, the proportion of drug tolerant *Mtb* increases, leading to higher minimal inhibitory concentrations or minimum bactericidal concentrations for several antibiotics (Wayne and Hayes, 1996; Deb et al., 2009; Gengenbacher et al., 2010; Larrouy-Maumus et al., 2016; Sarathy et al., 2018; Baker and Abramovitch, 2018; Xie et al., 2005). These data support that exposure to host derived stresses contributes to the increased *Mtb* antibiotic tolerance observed during infection.

*Mtb* stress responses are complex and involve multiple transcriptional, proteomic, and metabolic changes aimed at promoting pathogen survival. The resulting increase in drug tolerance that emerges in these conditions is indisputably multifactorial. Recent reviews have focused on the role of transcriptional adaptation (Kundu and Basu, 2021), the stringent response (Sharma et al., 2021), bacterial respiration (Hasenoehrl et al., 2021), and drug efflux in *Mtb* drug tolerance (Remm et al., 2021). In this review, we will focus on the role of fluctuations in central carbon metabolism in promoting drug tolerance of *Mtb* and discuss how continued dissection of the link between central carbon metabolism and drug tolerance will provide novel therapeutic approaches to target drug tolerant *Mtb*.

## Carbon metabolism in *Mtb*

**Figure 1:**
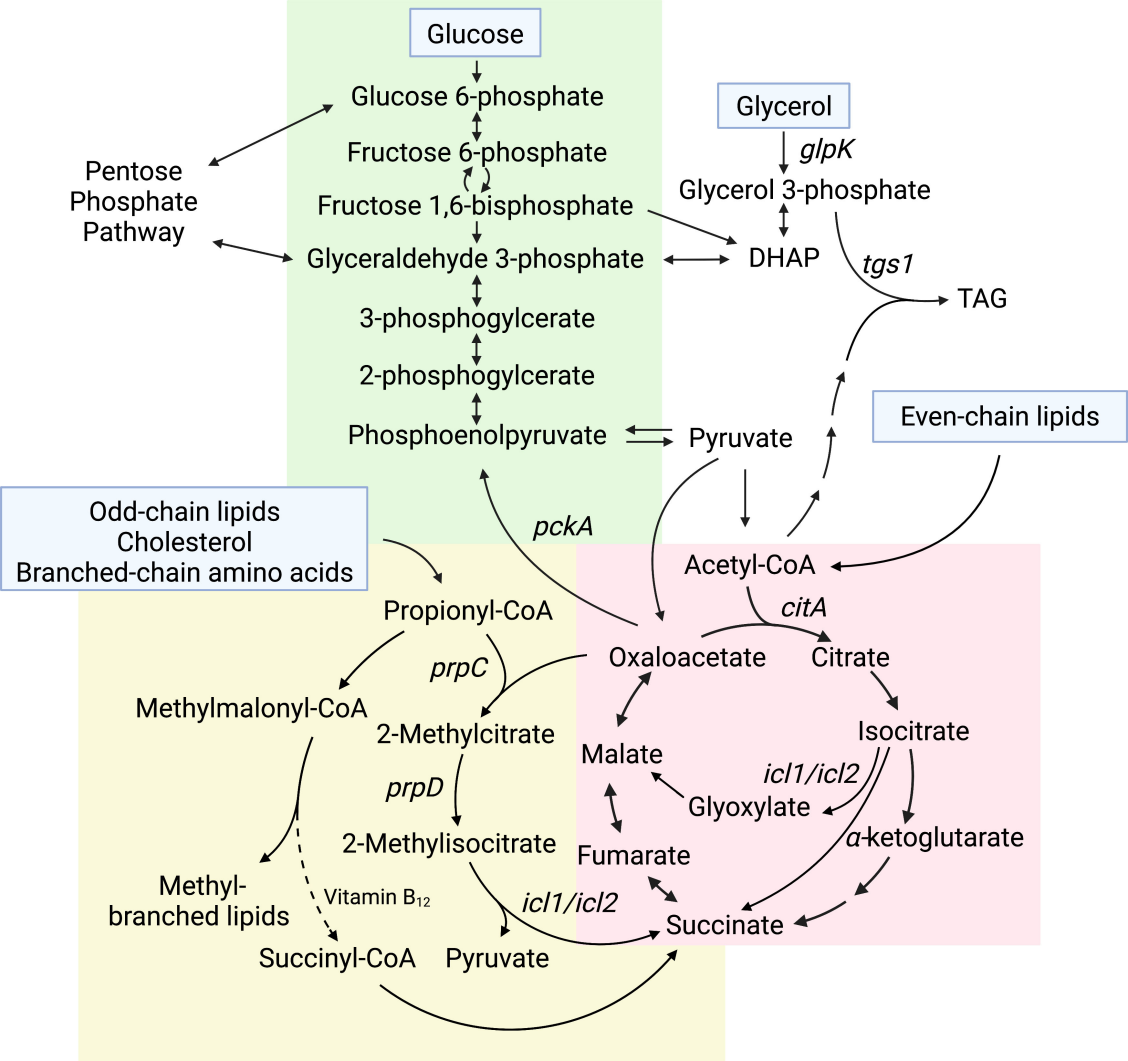
Core central carbon metabolism pathways that impact *Mtb* drug sensitivity. Carbon sources that feed into *Mtb* central carbon metabolism are listed in blue boxes. Carbon flowing down the pathway from glucose toward pyruvate, indicated by the downward pointing arrows, is glycolysis, whereas the reverse pathway, indicated by upward pointing arrows, is gluconeogenesis (green background). Even-chain lipids feed into the TCA cycle (red background). Odd-chain lipids, cholesterol, and branched-chain amino acids feed into the MCC and methylmalonyl-CoA pathways (yellow background). Genes discussed in the text that impact antibiotic sensitivity are indicated on the pathway.

*Mtb* grown *in vitro* can metabolize multiple carbon sources, even simultaneously (de Carvalho et al., 2010). Some of the most common carbon sources used to culture *Mtb* include glucose, glycerol, and oleic acid (Larsen et al., 2007). Glucose and other sugars are metabolized primarily through glycolysis and the pentose phosphate pathway to generate ATP and reducing equivalents (**Figure 1**). Glycerol is also used to generate ATP and reducing equivalents through glycolysis, or it can be anabolized *via* gluconeogenesis to synthesize sugars. To assimilate into these pathways, glycerol must first be converted to glycerol-3-phosphate by GlpK and then oxidized to dihydroxyacetone phosphate (DHAP)(**Figure 1**). Oleic acid and other even-chain fatty acids are catabolized to acetyl-CoA, which enters the tricarboxylic acid (TCA) cycle (**Figure 1**). The TCA cycle is critical for the generation of the reducing equivalents NADH and NADPH, as well as biosynthetic precursors for multiple other pathways, including synthesis of several amino acids. In particular, α-ketoglutarate can be converted to glutamate, which is a precursor for glutamine, arginine, and proline synthesis, and oxaloacetate can be converted to aspartate, which serves as a precursor for the synthesis of several amino acids including asparagine, methionine, lysine, threonine, and isoleucine. Mutants that are auxotrophic for one or more of these amino acids, including glutamine (Lee et al., 2006), arginine (Gordhan et al., 2002), aspartate (Jansen et al., 2020), methionine (Berney et al., 2015; Hasenoehrl et al., 2019), lysine (Pavelka et al., 2003), and threonine (Hasenoehrl et al., 2019) are severely attenuated during infection, demonstrating that the ability to synthesize these amino acids from TCA cycle intermediates is critical for *Mtb* to establish and maintain infection in the host. The essentiality of de novo amino acid biosynthesis during infection is particularly surprising because *Mtb* can assimilate nitrogen from asparagine, aspartate, glutamate, glutamine, leucine, alanine, and glycine during growth in macrophages *in vitro* (Gouzy et al., 2014; Borah et al., 2019). *Mtb* can also divert carbon from the CO2-generating steps of the TCA cycle *via* the glyoxylate shunt pathway (Muñoz-Elías and Mckinney, 2005). The glyoxylate shunt enables growth on fatty acids as a sole carbon source because it prevents loss of carbon *via* CO2, allowing for net gain of carbon from acetyl-CoA. This carbon can then be routed to other essential biosynthetic pathways such as amino acid synthesis or gluconeogenesis to generate cell wall precursors. In contrast, carbon sources that feed into glycolysis can be used to re-generate TCA cycle intermediates, allowing for carbon to leave the TCA cycle for biosynthesis and also be replenished independent of the glyoxylate shunt.

As opposed to *in vitro* cultures where *Mtb* can utilize multiple different carbon sources, *Mtb* isolated directly from infected mouse lungs was found to preferentially metabolize fatty acids over other carbon sources such as glucose or glycerol (Segal and Bloch, 1956). In humans, direct RNA-sequencing of *Mtb* from patient sputum revealed up-regulation of transcripts encoding enzymes required for cholesterol degradation (Lai et al., 2021). Furthermore, the Mtb-specific cholesterol byproduct 4-cholesten-3-one is increased in patients with active tuberculosis, suggesting that *Mtb* actively metabolizes cholesterol during infection (Chandra et al., 2022). These data indicate that *Mtb* carbon metabolism is shifted in the host to preferentially rely on lipids over carbohydrate carbon sources.

The preferential use of lipids by *Mtb* during infection is further supported by experiments using *Mtb* mutants in metabolic pathways, which demonstrate that *Mtb* requires the glyoxylate shunt to colonize mice and requires cholesterol uptake and catabolism to maintain infection (Muñoz-Elías and Mckinney, 2005; Pandey and Sassetti, 2008; Nesbitt et al., 2010). This is consistent with data showing that gluconeogenesis, which allows TCA cycle intermediates to be used to generate essential cell wall precursors, is more important than glycolysis for *Mtb* growth in the host. While a mutant that lacks hexose kinase activity, the first step of glycolysis, is only slightly attenuated later during infection (Marrero et al., 2013), mutants lacking enzymes required for gluconeogenesis are unable to grow in mice at all (Marrero et al., 2013; Puckett et al., 2014; Trujillo et al., 2014; Ganapathy et al., 2015). These findings demonstrate that *Mtb* relies on gluconeogenic substrates, such as lipids, for growth during infection, rather than sugars or glycerol. Therefore, the host environment, which induces a higher proportion of drug tolerant *Mtb*, also leads to a shift in *Mtb* metabolic requirements compared to unstressed *in vitro* culturing conditions.

## The impact of lipid metabolism in *Mtb* on drug tolerance

Triacylglycerols (TAG) and cholesterol are abundant lipid carbon sources available to *Mtb* during infection (Kim et al., 2010). *Mtb* liberates free fatty acids from TAG Deb et al.,2006, which are oxidized to acetyl-CoA, and degrades cholesterol through a series of reactions to pyruvate, acetyl-CoA, succinyl-CoA, and propionyl-CoA (Wilburn et al., 2018). The majority of cholesterol degradation products can directly feed into the TCA cycle or serve as substrates for gluconeogenesis. The exception is propionyl-CoA, which is toxic to the bacteria if it is not metabolized further (Munoz-Elias et al., 2006; Eoh and Rhee, 2014). Propionyl-CoA can be coupled with oxaloacetate through the methylcitrate cycle (MCC) to be detoxified to succinate and pyruvate (**Figure 1**) (Munoz-Elias et al., 2006; Eoh and Rhee, 2014). However, the MCC is dispensable for infection (Munoz-Elias et al., 2006), which may be because the environment encountered in the host enables propionyl-CoA detoxification through two alternative pathways. Specifically, the presence of exogenous even-chain fatty acids would enable *Mtb* to detoxify propionyl-CoA through incorporation into methyl-branched lipids, and access to vitamin B12 would enable detoxification of propionyl-CoA to succinyl-CoA (Jain et al., 2007; Savvi et al., 2008; Lee et al., 2013). Therefore, access to lipids or to vitamin B12 may obviate the need for the MCC during growth in the host even though the bacteria are catabolizing cholesterol.

**Figure 2:**
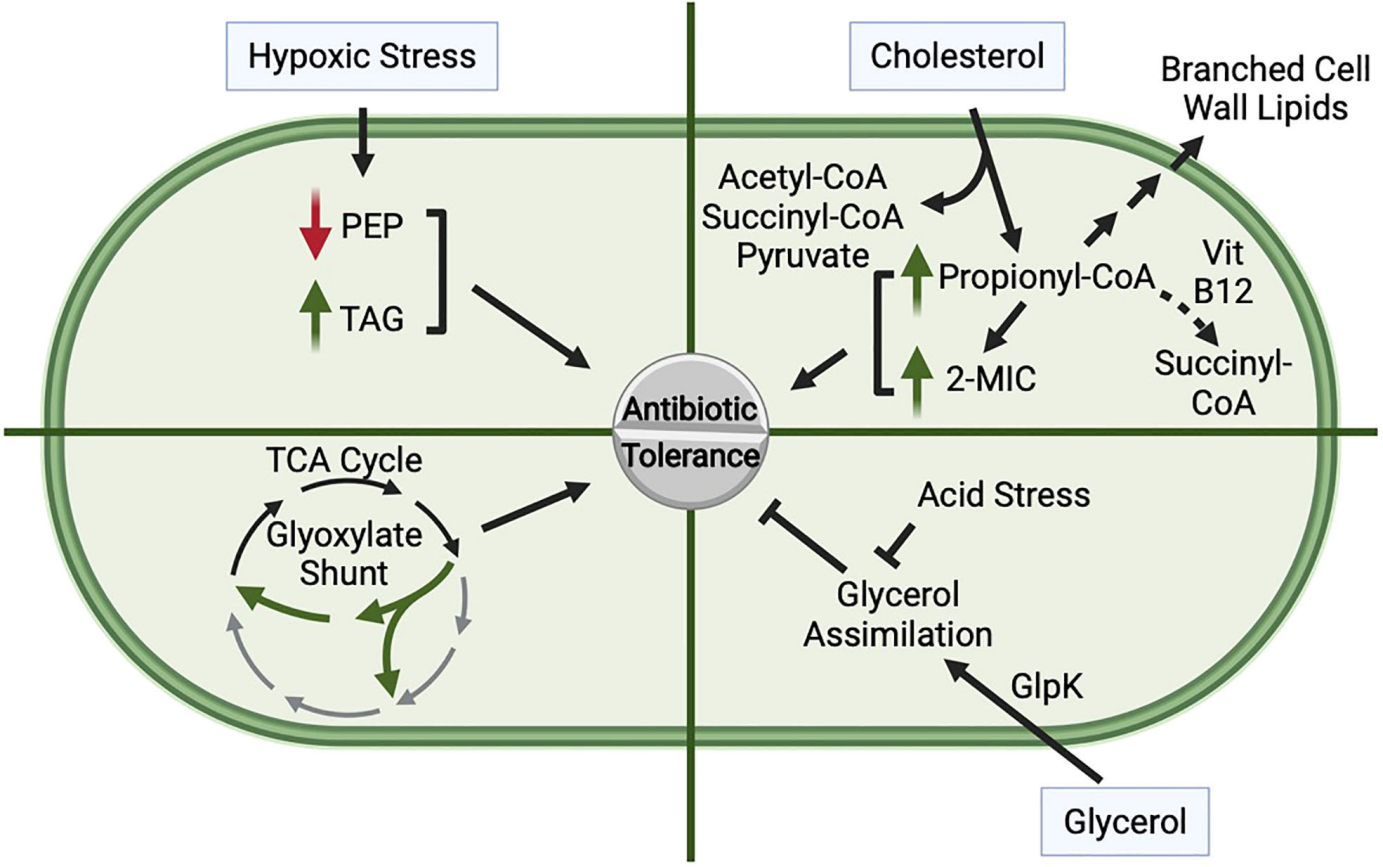
Role of Central Carbon Metabolism in Promoting Antibiotic Tolerance. Hypoxic stress, cholesterol metabolism, glycerol assimilation, low pH, and shunting of the TCA cycle *via* the glyoxylate shunt can each impact antibiotic tolerance of *Mtb.* Hypoxia: Exposure of *Mtb* to hypoxia leads to decreased levels of phosphoenolpyruvate (PEP) and an accumulation of triacylglycerol (TAG), both of which lead to an increase in drug tolerance. Cholesterol: Cholesterol is catabolized to acetyl-CoA, succinyl-CoA, pyruvate, and propionyl-CoA. Propionyl-CoA is detoxified through multiple pathways, including assimilation into branched chain lipids, conversion to succinyl-CoA through a vitamin B12-dependent pathway, or through the methylcitrate cycle (MCC) in which methylisocitrate (2-MIC) is an intermediate. Supplementation with cholesterol, propionate, or 2-MIC promotes antibiotic tolerance, and mutant strains that accumulate elevated levels of propionyl-CoA or 2-MIC are more tolerant to antibiotics. Glycerol Assimilation: Glycerol is assimilated into glycolysis and gluconeogenesis through phosphorylation by GlpK. Loss of glycerol catabolism leads to increased drug tolerance, suggesting that glycerol assimilation antagonizes antibiotic tolerance. Furthermore, *Mtb* in low pH is unable to efficiently catabolize glycerol, likely due to defects in glycolysis, resulting in increased antibiotic tolerance. Glyoxylate Shunt Activity: Mutants that lack the glyoxylate shunt are more sensitive to antibiotics, suggesting that rerouting carbon through the glyoxylate shunt promotes antibiotic tolerance.

Metabolism of cholesterol and the production of propionyl-CoA are associated with increased *Mtb* drug tolerance (**Figure 2**). *Mtb* grown in media containing cholesterol as a sole carbon source or containing mixed carbon sources including propionate exhibits decreased sensitivity to rifampicin (Koh et al., 2022). Exposure to propionate also activates PrpR, a regulator that induces expression of the *prpDC* operon, which encodes MCC enzymes PrpD and PrpC (**Figure 1**) (Masiewicz et al., 2012). Mutants with reduced or no PrpR activity, which are presumed to accumulate propionyl-CoA due to decreased expression of *prpDC*, exhibit slower growth in media containing propionate and increased tolerance to isoniazid, rifampicin, and ofloxacin (Hicks et al., 2018). Supplementing the *prpR* mutants with vitamin B12 enables shunting of propionyl-CoA to succinyl-CoA *via* methylmalonyl-CoA and is sufficient to rescue the growth defect in propionate media and reverse the drug tolerance of the mutants, supporting that accumulation of MCC intermediates contributes to drug tolerance (Hicks et al., 2018). The prpR mutants are similarly less sensitive to killing by antibiotics during *in vitro* infection of human macrophages (Hicks et al., 2018). Consistent with a role for PrpR-mediated regulation of the MCC in drug tolerance, mutations in prpR were enriched in drug resistant clinical isolates (Hicks et al., 2018). Since prpR mutations are associated with but do not confer drug resistance, it is possible these mutations promote a drug tolerance phenotype during infection, allowing the bacteria to survive and subsequently acquire drug resistance mutations. Consistent with decreased MCC activity promoting drug tolerance, knocking down expression of Icl1, which performs the final enzymatic step in the MCC, leads to accumulation of MCC intermediates in *Mtb* cultured in propionate media and causes nearly 10-fold less killing by isoniazid (Quinonez et al., 2022). In addition, exposure of *Mtb* to exogenous methylisocitrate, an MCC intermediate, is sufficient to decrease killing by isoniazid (Quinonez et al., 2022). Collectively, these studies suggest that accumulation of propionyl-CoA or MCC intermediates leads to antibiotic tolerance.

Propionyl-CoA accumulation slows *Mtb* growth and slow growth rate has been associated with drug tolerance (Wayne and Hayes, 1996; Baek et al., 2011). Therefore, reduced growth rate could explain how cholesterol metabolism decreases antibiotic sensitivity. Another possible contributor to cholesterol-induced drug tolerance could be the changes to cell wall lipids that occur during propionyl-CoA metabolism. Growth on propionyl-CoA-generating carbon sources causes *Mtb* to synthesize branched lipids such as phthiocerol dimycocerosate (PDIM) and sulfolipid-1 with increased chain lengths (Jain et al., 2007; Yang et al., 2009; Griffin et al., 2012; Borah et al., 2021; Koh et al., 2022). PDIM is a major structural lipid intercalated in the outer *Mtb* envelope and has been shown to create a barrier that is particularly impenetrable to polar molecules (Wang et al., 2020). Thus, it is possible that alterations to PDIM chain length during growth on cholesterol or propionate may impact the permeability of the *Mtb* cell envelope, which could explain the altered antibiotic sensitivity.

## *Mtb* metabolism during hypoxia and the association with drug tolerance

During exposure to hypoxia, *Mtb* exhibits decreased levels of phosphoenolpyruvate (PEP) (**Figure 2**), an intermediate in glycolysis and gluconeogenesis, which is likely caused by decreased synthesis of PEP from oxaloacetate (Lim et al., 2021) (**Figure 1**). Supplementing hypoxic *Mtb* with exogenous PEP enhances killing by isoniazid (Lim et al., 2021), suggesting that the decrease in PEP during hypoxia contributes to hypoxia-induced drug tolerance. Notably, supplementation with pyruvate does not have the same effect, suggesting that this effect is specific for PEP, and access to additional carbon alone is not sufficient to sensitize *Mtb* to isoniazid. PEP supplementation also promotes *Mtb* sensitivity to D-cycloserine, a cell wall biosynthesis inhibitor, in aerated conditions, suggesting that the effect of PEP on drug tolerance is not specific for hypoxia (Lim et al., 2021). In addition to feeding into glycolysis and/or gluconeogenesis, PEP can also feed into the TCA cycle by conversion into oxaloacetate, can serve as a substrate for synthesis of the peptidoglycan precursor N-acetylmuramic acid, and is a substrate for the shikimate pathway (Lim et al., 2021). Which of these pathways contributes to the PEP-dependent drug sensitivity is still unknown.

During hypoxia, there is also decreased flux through several NAD(P)H-generating steps of the TCA cycle, likely to prevent production of NAD(P)H in conditions that these cofactors cannot be re-oxidized. This altered flux is caused by re-routing of acetyl-CoA to fatty acid and subsequent TAG biosynthesis (Baek et al., 2011), increased glyoxylate shunt activation (Eoh and Rhee, 2013), and reversal of several steps in the TCA cycle to generate succinate from oxaloacetate (Watanabe et al., 2011; Zimmermann et al., 2015). Deletion of the TAG biosynthesis gene *tgs1* or overexpression of the citrate synthase gene *citA* prevented re-rerouting of acetyl-CoA to fatty acid and TAG biosynthesis during hypoxia and iron starvation (Baek et al., 2011). These mutants failed to arrest growth and exhibited enhanced sensitivity to isoniazid, streptomycin, ciprofloxacin, and ethambutol in hypoxic and iron starvation conditions (Baek et al., 2011). The Δ*tgs1* and *citA*-overexpressing strains were also significantly more sensitive to killing by isoniazid in a mouse model of infection, supporting that the re-routing of acetyl-CoA to fatty acid biosynthesis promotes drug tolerance (**Figure 2**) (Baek et al., 2011). Redirecting carbon away from the TCA cycle can also promote drug tolerance in aerated conditions. The glyoxylate shunt enables bypassing of two NAD(P)H- and CO2-generating steps of the TCA cycle. In addition to promoting growth on lipids by conserving carbon, the glyoxylate shunt may also decrease the generation of NAD(P)H during growth on glucose, preventing oxidative stress caused by electron transport chain activity (Nandakumar et al., 2014). A Δ*icl1/icl2* double mutant, which lacks the first step of the glyoxylate shunt, exhibits >10-fold enhanced killing by isoniazid, rifampicin, or streptomycin compared to the wild-type strain during aerobic growth on glucose (Nandakumar et al., 2014). Therefore, diverting carbon through the glyoxylate shunt can promote *Mtb* drug tolerance (**Figure 2**), likely by alleviating oxidative stress caused by TCA cycle and downstream electron transport chain activity. These studies demonstrate that by re-routing carbon away from the TCA cycle, *Mtb* becomes more tolerant to antibiotics.

## Glycerol metabolism and low pH-induced drug tolerance

*Mtb* is unable to grow on glycerol as the sole carbon source in low pH (Baker et al., 2014). This is likely due to inefficient assimilation of glycerol into lower glycolysis caused by decreased glyceraldehyde-3-phosphate dehydrogenase activity in low pH (Gouzy et al., 2021) (**Figure 1**). This nonpermissive growth condition results in acidic pH-induced drug tolerance (**Figure 2**), whereas growth on pyruvate, which enables *Mtb* growth in low pH, prevents the pH-induced drug tolerance (Baker and Abramovitch, 2018). Glycerol is assimilated into glycolysis and gluconeogenesis through phosphorylation by GlpK and subsequent conversion to DHAP (**Figure 1**). In media containing glycerol and other carbon sources, a Δ*glpK* mutant exhibited decreased sensitivity to isoniazid and rifampicin compared to wild-type *Mtb*, further supporting that decreasing glycerol metabolism increases drug tolerance (Bellerose et al., 2019; Safi et al., 2019). *glpK* is dispensable in the mouse model of *Mtb* infection, suggesting that glycerol is not a primary carbon source in mice (Pethe et al., 2010). However, free glycerol is detectable in infected mouse lungs, suggesting *Mtb* would have access to glycerol in the host (Safi et al., 2019). Furthermore, a Δ*glpK* mutant survived better than wild-type *Mtb* during treatment with pyrazinamide or any drug combination involving pyrazinamide, but not during isoniazid or rifampicin monotherapy, in a mouse model of infection (Bellerose et al., 2019). Therefore, the inability to metabolize glycerol during infection in mice promotes *Mtb* drug tolerance specifically to pyrazinamide. Since the *glpk* mutant does not exhibit increased tolerance to pyrazinamide *in vitro*, the pyrazinamide-specific tolerance in the *glpK* mutant is dependent upon the microenvironment within the host (Bellerose et al., 2019). Multiple groups have also identified *glpK* mutations in *Mtb* clinical isolates (Bellerose et al., 2019; Safi et al., 2019; Vargas and Farhat, 2020), and in some datasets these mutants are more commonly found in drug resistant isolates than in drug sensitive isolates (Bellerose et al., 2019; Safi et al., 2019). The *glpK* mutations are associated with but do not confer drug resistance themselves. However, if these mutations promote drug tolerance, they may enable *Mtb* to survive during antibiotic therapy, extending the time wherein *Mtb* may acquire a drug resistance mutation.

## Drug tolerance is often conditional

In this review, we have highlighted studies demonstrating that *Mtb* undergoes changes in carbon metabolism in response to host derived stresses that render *Mtb* more tolerant to antibiotics. However, the observed drug tolerance is rarely pan-antibiotic. For example, in hypoxic conditions, while *Mtb* becomes extremely tolerant to some antibiotics, including isoniazid, rifampicin, and streptomycin, it remains susceptible to antibiotics that target ATP synthase, and in some cases becomes more sensitive to killing by ATP synthase inhibitors and other antibiotics that target the electron transport chain (Koul et al., 2008; Rao et al., 2008; Gengenbacher et al., 2010; Sarathy et al., 2018; Lee et al., 2021). Furthermore, in an experiment to identify *Mtb* mutants with altered sensitivity to either isoniazid, rifampicin, pyrazinamide, or ethambutol during mouse infection, the majority of mutants identified only exhibited significantly altered susceptibility to a single antibiotic (Bellerose et al., 2020). Therefore, antibiotic tolerance can be conditional and specific to individual antibiotics.

Metabolic changes in *Mtb* that result in increased drug tolerance are often correlated with growth arrest, including toxicity mediated by propionyl-CoA or MCC intermediate accumulation (Hicks et al., 2018; Quinonez et al., 2022), hypoxia-induced TAG accumulation (Baek et al., 2011), and decreased glycerol metabolism in low pH conditions (Baker et al., 2014). However, there is also data, particularly in infection models, that suggests growth arrest is not universally associated with antibiotic tolerance (Raffetseder et al., 2014; Bellerose et al., 2019, Bellerose et al., 2020). Specifically, the Δ*glpK* mutant had no fitness defect in mice yet had altered antibiotic susceptibility to pyrazinamide (Bellerose et al., 2019). In addition, although there is a correlation between mutants that were less sensitive to isoniazid and mutants that had a fitness defect in mice, this association was not observed with mutants that were less sensitive to the other antibiotics (Bellerose et al., 2020).

## Conclusion

Understanding the mechanisms by which *Mtb* metabolism impacts tolerance to specific antibiotics, particularly in the host environment, could lead to novel therapeutic approaches. We have highlighted several studies that demonstrate it is possible to manipulate metabolic pathways to reverse tolerance to a number of frontline antibiotics. For example, the *Mtb* Δ*tgs1* mutant is more susceptible to killing by isoniazid, rifampicin, or streptomycin during stress and in mice (Baek et al., 2011). This suggests that developing inhibitors of TAG biosynthesis could be a viable approach to enhance the efficacy of these antibiotics in the clinic. Additionally, supplementation with exogenous PEP was sufficient to enhance killing of hypoxic *Mtb* by isoniazid (Lim et al., 2021). Thus, changing the metabolic state of *Mtb* can potentiate killing by frontline antibiotics. Our review focused on central carbon metabolism, however these pathways are intricately connected to other metabolic networks, such as amino acid biosynthesis. De novo biosynthesis of amino acids from the TCA cycle and other pathways is essential for *Mtb* virulence and recent studies have shown that inhibiting amino acid biosynthesis is a promising approach for therapeutic development (Wellington et al., 2017). Although some metabolic enzymes may not be druggable targets due to shared structural homology with the mammalian homolog or difficulty in identifiying small molecules that effectively inhibit the enzyme, further elucidation of which metabolic pathways are essential during infection and how specific pathways contribute to drug tolerance will provide new opportunities for exploration.

## Author contribution

All authors participated in the conceptualization and writing of this mini-review.

## Funding

CS is supported by NIH grant AI134847 as well as a Burroughs Wellcome Fund Investigators in the Pathogenesis of Infectious Disease Award. GH is supported by National Science Foundation graduate research fellowship DGE-1745038 and NIGMS cell and molecular biology training grant GM007067. EW is supported by NIAID award T32AI007172. The content is solely the responsibility of the authors and does not necessarily represent the official views of the National Institutes of Health.

## Acknowledgments

We are grateful to H. Eoh for kindly sharing his unpublished manuscript for this issue while we prepared our final versions.

## Conflict of interest

The authors declare that the research was conducted in the absence of any commercial or financial relationships that could be construed as a potential conflict of interest.

## Publisher’s note

All claims expressed in this article are solely those of the authors and do not necessarily represent those of their affiliated organizations, or those of the publisher, the editors and the reviewers. Any product that may be evaluated in this article, or claim that may be made by its manufacturer, is not guaranteed or endorsed by the publisher.
